# Failure of Empirical Antimicrobial Therapy in Diabetic Foot Ulcer With Chronic Osteomyelitis: A Case Report

**DOI:** 10.7759/cureus.95492

**Published:** 2025-10-27

**Authors:** Melike Karabulut Ozer

**Affiliations:** 1 Department of Family Medicine, Ordu University Faculty of Medicine, Ordu, TUR

**Keywords:** diabetic foot infection, empirical therapy, multi-drug resistance, osteomyelitis, ­wound healing

## Abstract

Diabetic foot infections (DFIs) are frequent and severe complications of diabetes mellitus that can lead to chronic wounds, osteomyelitis, and even limb amputation if not properly managed. This case report describes the clinical consequences of prolonged empirical antibiotic therapy without culture guidance in a patient with a diabetic foot abscess. A 72-year-old man with type 2 diabetes and hypertension presented with a chronic, non-healing deep foot abscess after receiving 15 consecutive courses of empirical oral antibiotics over eight months. Magnetic resonance imaging suggested osteomyelitis of the fifth metatarsal bone. Urgent surgical drainage and tissue cultures revealed *Escherichia coli* and *Pseudomonas aeruginosa*, both resistant to multiple commonly used antibiotics but susceptible to ceftriaxone and carbapenems. Targeted intravenous therapy with ceftriaxone and metronidazole led to rapid improvement within one week and complete healing by the third month. This case emphasizes that prolonged empirical therapy may lead to treatment failure and antimicrobial resistance. Early surgical intervention combined with culture-guided antibiotic selection is crucial for effective management of DFIs and for preventing severe complications such as amputation. This case underscores the importance of multidisciplinary and microbiologically guided approaches.

## Introduction

Diabetic foot infections (DFIs) are among the most common and devastating complications of diabetes mellitus, significantly increasing morbidity, healthcare costs, and amputation risk [1,2]. DFIs are often polymicrobial and involve multidrug-resistant organisms (MDROs), complicating management and prolonging healing [3,4]. Empirical antibiotic therapy is a widespread initial approach; however, prolonged use without microbiological confirmation can select resistant strains, delay appropriate therapy, and worsen outcomes [5,6].

Recent evidence demonstrates that empirical antibiotic therapy without culture guidance significantly increases hospitalization rates, with patients receiving empirical treatment showing a higher risk for adverse outcomes compared to culture-directed therapy [7]. The pathophysiology of DFIs involves complex interactions between bacterial virulence factors and host immune dysfunction. Biofilm formation represents a critical mechanism that significantly contributes to treatment failure, with studies indicating that biofilm-forming organisms demonstrate significantly enhanced antibiotic resistance [8].

Current international guidelines emphasize the importance of early microbiological sampling and culture-guided antibiotic therapy to optimize treatment outcomes [9]. However, challenges remain in distinguishing between infection and colonization, particularly in polymicrobial environments. This case report illustrates the potential pitfalls of extended empirical therapy and highlights the importance of early surgical drainage and culture-guided antibiotic therapy in the management of diabetic foot abscesses.

## Case presentation

**Figure 1:**
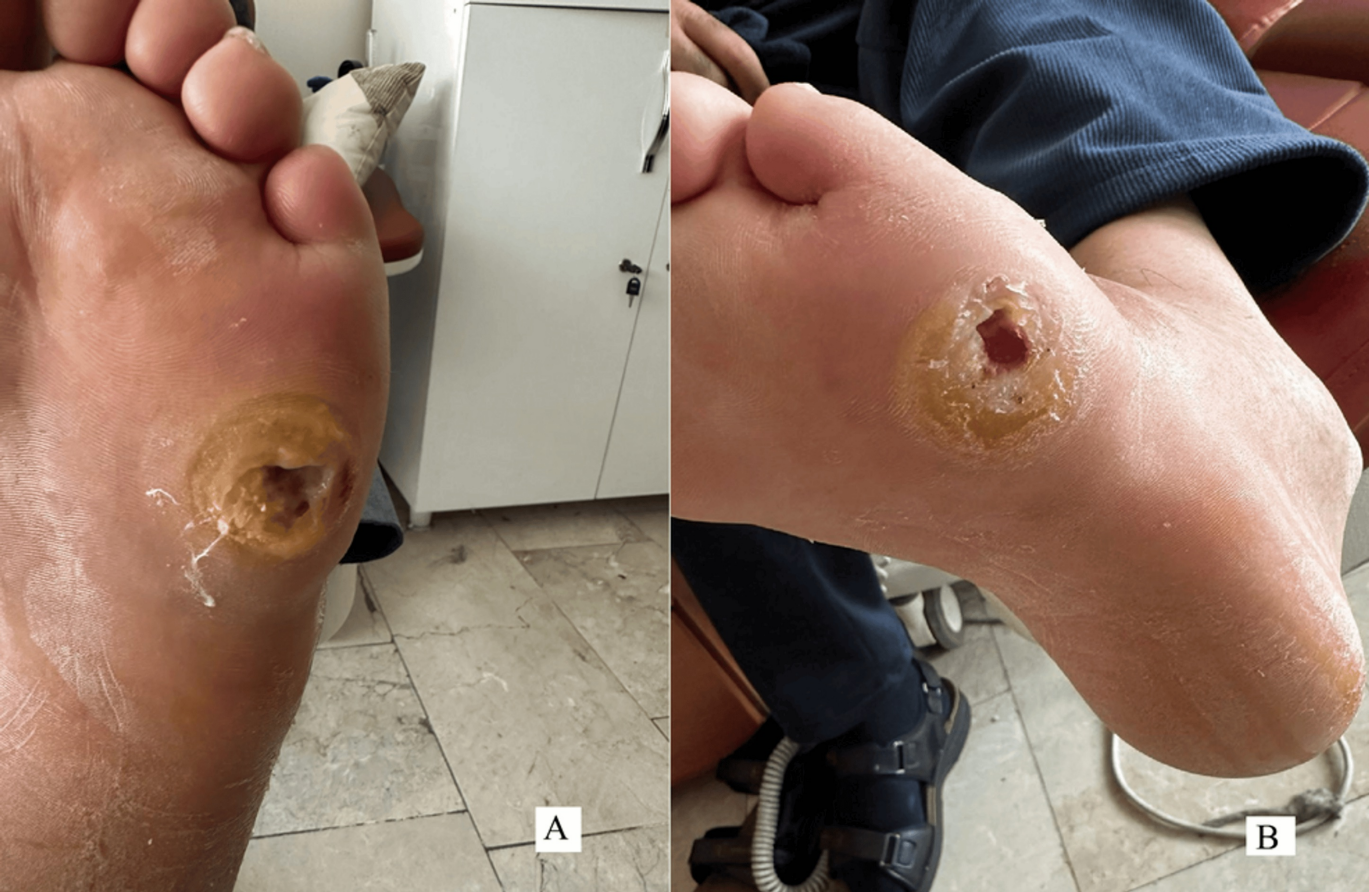
Clinical progression of the diabetic foot abscess (A) Initial clinical appearance of the diabetic foot at presentation, demonstrating erythema, swelling, and purulent discharge; (B) After one week of targeted intravenous antibiotic therapy, showing reduced inflammation and granulation tissue formation.

A 72-year-old man with a 15-year history of type 2 diabetes mellitus and hypertension presented with a chronic, painful ulcer on the right foot. The lesion measured 3×3 cm, extended from the plantar to the dorsal aspect, and exhibited erythema, swelling, and purulent discharge (Figure 1A).

The ulcer was classified as Wagner grade 3 [10]. Over the preceding eight months, the patient had received multiple empirical oral antibiotic courses at different facilities, most recently cefuroxime axetil 500 mg twice daily and trimethoprim-sulfamethoxazole 800/160 mg twice daily, without any improvement. Vascular evaluation by arterial Doppler ultrasound revealed no significant pathology, and an ankle-brachial index (ABI) was not performed.

**Figure 2:**
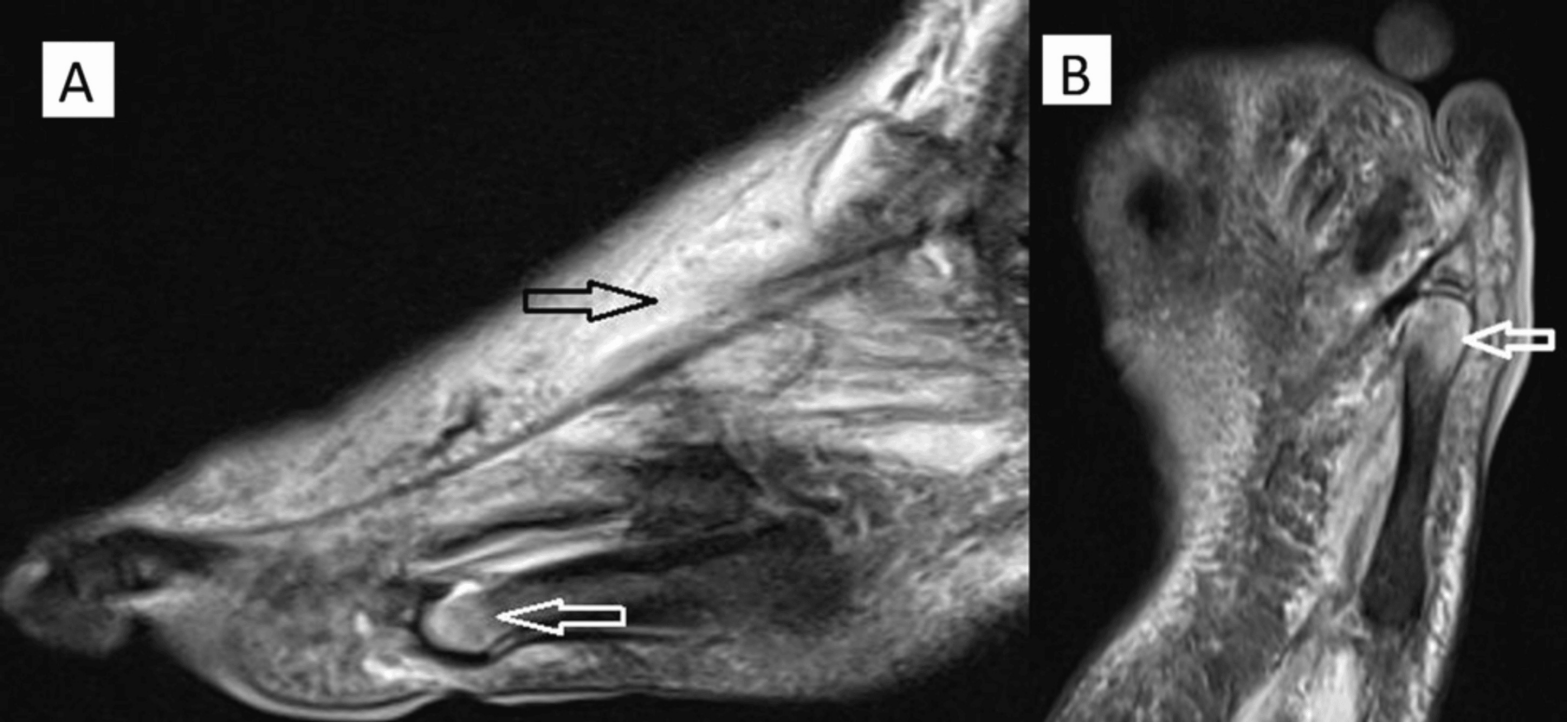
Magnetic resonance imaging findings of diabetic foot osteomyelitis (A) Sagittal MRI image showing bone marrow edema consistent with osteomyelitis (white arrow) and soft tissue edema signal compatible with infection (black arrow); (B) Coronal MRI image demonstrating bone marrow edema consistent with osteomyelitis in the fifth metatarsal head (white arrow).

On March 12, 2025, urgent surgical debridement and abscess drainage were performed, and deep tissue samples were obtained for microbiological analysis. Magnetic resonance imaging (MRI) performed at that time demonstrated hyperintense signal changes consistent with bone marrow edema, indicating osteomyelitis of the fifth metatarsal head (Figure 2A), along with adjacent soft-tissue edema (Figure 2A) and coronal confirmation of bone marrow involvement (Figure 2B).

Baseline laboratory tests on the same day showed HbA1c of 8.0% (reference: <5.7%), white blood cell (WBC) count of 9.15 × 10^9^/L (reference: 4-10 ×10^9^/L), and CRP of 83.41 mg/L (reference: <5 mg/L).

The patient was admitted on March 16, 2025, and intravenous ceftriaxone 1 g every 12 hours plus metronidazole 500 mg every 12 hours was initiated for seven days. Culture results from the debridement tissue yielded *Escherichia coli *and *Pseudomonas aeruginosa*. *E. coli *was resistant to ampicillin and trimethoprim-sulfamethoxazole but susceptible to ceftriaxone, ceftazidime/cefepime, piperacillin-tazobactam, carbapenems, and aminoglycosides. *P. aeruginosa* was susceptible to piperacillin-tazobactam (high dose), ceftazidime (high dose), ceftolozane-tazobactam, and amikacin, but resistant to cefepime and fluoroquinolones.

Despite the isolation of Pseudomonas, the patient showed early and marked clinical improvement within one week under ceftriaxone + metronidazole following surgical source control, and therefore, treatment was not broadened in line with antimicrobial stewardship principles (Figure 1B).

**Figure 3:**
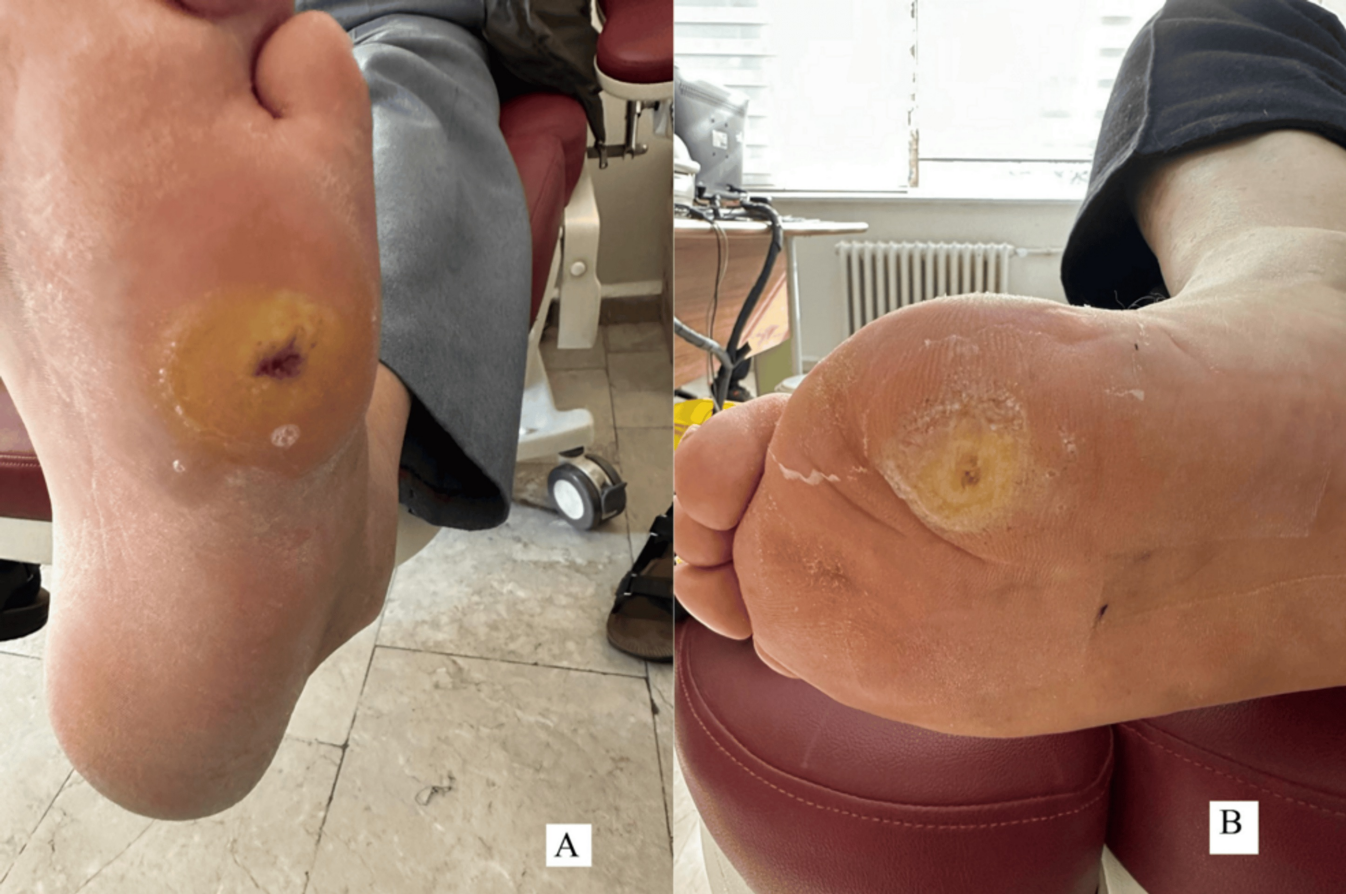
Progressive healing of the foot ulcer to complete epithelization (A) One month after treatment, showing significant ulcer size reduction and healthy granulation tissue; (B) Three months after treatment, demonstrating complete epithelialization with minimal residual hyperkeratosis.

At one month, the ulcer had decreased to 1×1 cm with healthy granulation tissue (Figure 3A), and by June 26, 2025 (after approximately three months), it was completely epithelialized with mild residual hyperkeratosis (Figure 3B).

No oral step-down therapy was prescribed, and no recurrence was observed during follow-up.

## Discussion

This case demonstrates that prolonged empirical therapy can fail in DFIs, particularly in polymicrobial infections involving resistant pathogens [3,4,11]. Studies have reported that MDROs are isolated in 30-70% of diabetic foot ulcers, increasing treatment complexity and amputation risk [6,12]. *P. aeruginosa* and *E. coli *are frequently identified and often display resistance to empirical regimens, delaying effective therapy [13].

Early and appropriate microbiological evaluation is critical in DFIs. Empirical therapy may be justified initially, but should be re-evaluated if no clinical improvement is observed within a short period. Persistent use of ineffective antibiotics not only delays healing but also fosters the development of resistance [4,6]. Timely surgical drainage reduces bacterial load, removes necrotic tissue, and enhances antibiotic efficacy. The addition of anaerobic coverage, such as metronidazole, is often beneficial in deep or chronic infections where anaerobes may coexist [13].

This case supports current recommendations advocating early referral to specialized wound care clinics for non-healing diabetic foot ulcers [14]. Early culture-guided therapy improves outcomes, shortens healing time, and prevents severe complications, including limb amputation.

## Conclusions

This case highlights that empirical antimicrobial therapy may fail in chronic diabetic foot infections complicated by osteomyelitis, especially when polymicrobial flora and resistant organisms such as *P. aeruginosa* are involved. Despite initial empirical regimens, the absence of clinical response emphasizes the importance of obtaining deep tissue cultures and performing targeted antimicrobial therapy guided by microbiological data.

Moreover, early surgical intervention and adequate source control may have played an important role in the patient’s recovery. This observation suggests that surgical debridement combined with rational antibiotic use could represent an effective approach in similar cases. Although *P. aeruginosa *was isolated, the patient showed apparent clinical improvement following adequate debridement and treatment with a narrower-spectrum regimen (ceftriaxone + metronidazole). These findings indicate that therapeutic decisions should perhaps be guided not only by culture results but also by clinical judgment and principles of antimicrobial stewardship.
